# Nuclear insulin-like growth factor-1 receptor (IGF1R) displays proliferative and regulatory activities in non-malignant cells

**DOI:** 10.1371/journal.pone.0185164

**Published:** 2017-09-25

**Authors:** Ravid Solomon-Zemler, Rive Sarfstein, Haim Werner

**Affiliations:** 1 Department of Human Molecular Genetics and Biochemistry, Sackler School of Medicine, Tel Aviv University, Tel Aviv, Israel; 2 Yoran Institute for Human Genome Research, Tel Aviv University, Tel Aviv, Israel; Thomas Jefferson University, UNITED STATES

## Abstract

The insulin-like growth factor-1 receptor (IGF1R) mediates the biological actions of IGF1 and IGF2. The IGF1R is involved in both physiological and pathological activities and is usually overexpressed in most types of cancer. In addition to its classical mechanism of action, recent evidence has shown a nuclear presence of IGF1R, associated with novel genomic/transcriptional types of activities. The present study was aimed at evaluating the hypothesis that nuclear IGF1R localization is not restricted to cancer cells and might constitute a novel physiologically relevant regulatory mechanism. Our data shows that nuclear translocation takes place in a wide array of cells, including normal diploid fibroblasts. In addition, we provide evidence for a synergistic effect of a nuclear translocation blocker along with selective IGF1R inhibitors in terms of decreasing cell proliferation. Given the important role of the IGF1R in mitogenesis, the present results may be of translational relevance in cancer research. In conclusion, results are consistent with the concept that nuclear IGF1R fulfills important physiological and pathological roles.

## Introduction

The insulin-like growth factor-1 receptor (IGF1R) is a cell-surface receptor that belongs to the tyrosine kinase receptors super family [1]. Binding of the IGF1 or IGF2 ligands to the IGF1R extracellular domain activates the receptor catalytic domain and transmits defined signals through a number of intracellular substrates, including the insulin receptor substrate-1 (IRS-1) and Src homology collagen (Shc) proteins. These molecules, in turn, activate a cascade of protein kinases, including the phosphatidyl inositol-3 kinase (PI3K)-protein kinase B (PKB)/AKT and mitogen activated protein kinase (MAPK) signal transduction pathways [2–4]. These two major protein cascades control several biological processes, including transcription, apoptosis, cell growth and translation [5, 6].

In addition to its important role during development, there is evidence pointing to a pivotal role for IGF1R signaling in malignant transformation [7]. Activation of the cell-surface IGF1R by circulating or locally produced IGF1/IGF2 is a critical pre-requisite for transformation. Consequently, cells lacking IGF1R, for the most part, do not undergo transformation when exposed to oncogenic agents [8]. Clinical and experimental data collected over more than 30 years demonstrate that the vast majority of tumor cells display a large number of cell-surface IGF1Rs and express higher levels of IGF1R mRNA than normal cells [9]. In addition, ectopic overexpression of IGF1R in non-transformed cells led to a ligand-dependent, highly transformed phenotype, which included the formation of tumors in nude mice [7]. Hence, targeted therapies against the IGF1R (particularly blocking antibodies and tyrosine kinase inhibitors) emerged in recent years as a promising therapeutic approach in cancer treatment [10, 11].

Apart from the typical tyrosine kinase activity associated with IGF1R, our group and others have shown that the IGF1R can be modified by small ubiquitin-like modifier protein (SUMO)-1, with ensuing translocation to the nucleus [12–14]. Nuclear IGF1R was shown to act as a transcriptional activator, binding to specific genome regions in, apparently, a sequence-specific manner. Of interest, nuclear IGF1R was also shown to bind its cognate promoter and autoregulate promoter activity [12]. Furthermore, evidence has been presented showing that nuclear IGF1R binds to several transcription factors and co-activators, including transcription factor LEF1, leading to elevated levels of cyclin D1 and axin2, two important players in the cell cycle machinery [15].

Nuclear transport of cell-surface receptors, in general, and of the IGF1R in particular, constitutes a novel regulatory mechanism that may provide an additional layer of biological control. However, most experimental evidence so far was generated using cancer-derived cell lines as well as freshly obtained tumors or archival specimens. The question whether nuclear IGF1R translocation constitutes a common physiological process in normal, non-transformed cells, has not yet been explored in a systematic fashion. The present study was aimed at evaluating the hypothesis that nuclear IGF1R transport is not restricted to malignant cells and constitutes a novel physiologically relevant cellular mechanism. Our data shows that nuclear translocation takes place in a wide array of cells, including normal diploid fibroblasts. Nuclear IGF1R, hence, may provide an additional level of biological regulation in normal physiological processes.

## Materials and methods

### Cell cultures

The human non-malignant MCF10A breast cell line was maintained in DMEM F-12 medium (Biological Industries, Kibbutz Beit Haemek, Israel) supplemented with 5% horse serum, 100 microgram/ml EGF, 1 mg/ml cholera toxin, 10 mg/ml hydrocortisone and 10 mg/ml of insulin. Human breast cancer-derived MCF7 cells were maintained in Eagle's Minimum Essential Medium (EMEM; Biological Industries) supplemented with 10% fetal bovine serum (FBS) and 2 mM glutamine (Sigma-Aldrich, St. Louis, MO, USA). MCF10A and MCF7 cells were obtained from the American Type Culture Collection (Manassas, VA, USA). MCF7 cells with a silenced IGF1R (MCF7/IGF1R KO) were provided by Dr. Derek LeRoith (Rambam Medical Center, Haifa, Israel). The prostate cancer-derived P69 cell line was derived by immortalization of human primary prostate epithelial cells with simian virus-40 T antigen and is rarely tumorigenic. The M12 cell line was derived from the P69 cell line by selection for tumor formation in nude mice and is tumorigenic and metastatic. P69 and M12 cells were maintained in RPMI-1640 medium (Biological Industries) supplemented with 10% FBS and 2 mM glutamine. P69 and M12 cell lines were provided by Dr. Joy L. Ware (Medical College of Virginia, Richmond, VA, USA). Primary human fibroblasts, obtained from skin biopsies, were maintained in EMEM supplemented with 20% FBS and 2 mM glutamine. Primary human fibroblasts were kindly provided by Dr. Eli Sprecher (Sourasky Medical Center, Tel Aviv, Israel). All cells were cultured at 37°C in a humidified incubator with 5% CO_2_.

### Treatments

Cells were serum-starved for 24 hr, after which they were treated with 50 ng/ml of IGF1 (PeproTech Ltd., Rocky Hill, NJ) and/or 300 micromolar of dansylcadaverine (Sigma-Aldrich) for different time periods. In addition, cells were treated with the following specific IGF1R inhibitors: tyrosine kinase inhibitor NVP-AEW541 (2 micromolar) (Novartis) or tyrphostin AG1024 (1 micromolar) (Sigma-Aldrich).

### IGF1R knockdown with small interfering RNA (siRNA)

For siRNA knockdown experiments, siRNA against the human IGF1R [TCGAAGAATCGCATCATCATA-(Qiagen)] was used. Negative control [non-targeting (NT)] or siRNA against IGF1R were transfected using INTERFERin^TM^ (Polyplus Transfection, Illkirch, France). 10–15 nM of siRNA and 6 microliter of INTERFERin^TM^ were used for each transfection.

### Fractionation of cytoplasmic and nuclear proteins

Cells were lysed with harvest buffer (10 mM HEPES pH 7.9, 50 mM NaCl, 0.5 M sucrose, 0.1 mM EDTA, 0.5% Triton X-100, 1 mM DTT, 10 mM sodium pyrophosphate tetrabasic, 100 mM NaF, 17.5 mM beta-glycrophosphate, 1 mM PMSF, 4 microgram/ml aprotinin and 2 microgram/ml pepstatin A). The samples were centrifuged to pellet nuclei. The cytoplasmic fraction was transferred to a new tube. The nuclei was washed with buffer A (10 mM HEPES pH 7.9, 10 mM KCl, 0.1 mM EDTA, 0.1 mM EGTA, 1 mM DTT, 1 mM PMSF, 4 microgram/ml aprotinin and 2 microgram/ml pepstatin A) for 10 min. Nuclear proteins were lysed in buffer C (10 mM HEPES pH 7.9, 500 mM NaCl, 0.1 mM EDTA, 0.1 mM EGTA, 0.1% NP-40, 1 mM DTT, 1 mM PMSF, 4 microgram/ml aprotinin and 2 microgram/ml pepstatin A) for 15 min and transferred to a new tube, followed by the Western blot protocol.

### Western blot analyses

Samples were electrophoresed through 10% SDS-PAGE, followed by blotting of the proteins onto nitrocellulose membranes. After blocking with skim milk, the blots were incubated overnight with the antibodies listed below, washed, and incubated with the appropriate horseradish peroxidase (HRP)-conjugated secondary antibody. An antibody against tubulin (T5168) was purchased from Sigma-Aldrich. Antibodies against IGF1R (#3027), insulin receptor (IR) β-subunit (#3025), SP1 (#5931) and SUMO-1 (#2A12) were obtained from Cell Signaling Technology Inc (Beverly, MA, USA). Antibodies against lamin A/C (ab8984) and lamin B1 (ab16048) were purchased from Abcam (Cambridge, MA, USA). The secondary antibodies were goat anti-rabbit IgG (1:50,000) and donkey anti-mouse IgG (1:25,000; Jackson ImmunoResearch Laboratories, West Grove, PA, USA). Proteins were detected using the SuperSignal West Pico Chemiluminescent Substrate (Pierce, Waltham, MA, USA).

### Confocal microscopy analyses

Cells were cultured for 48 hr, after which they were washed with phosphate-buffered saline (PBS) and fixed on coverslips by using 4% paraformaldehyde in PBS for 20 min. Then, cells were blocked with 3% bovine serum albumin (BSA) + 0.5% TritonX-100 in PBS for 30 min at room temperature. Cells were then incubated with the IGF1R polyclonal antibody described above (1:100 dilution) for 2 hr at RT. Cells were washed with PBS and then incubated with a donkey anti-rabbit IgG at a 1:200 dilution (Alexa Fluor 488 # A21206) for an additional 30 min. Cells were washed with PBS and mounted on Vectashield mounting medium containing DAPI (Vector Laboratories Inc., Burlingame, CA). Staining was assessed using a laser-scanning confocal microscope (Leicasp5, Leica, Wetzlar, Germany).

### XTT cell proliferation assays

Cell proliferation was monitored using an XTT cell proliferation assay kit (Biological Industries), according to manufacturer’s protocol. MCF7, MCF10A, M12, P69, stable MCF7/IGF1R KO and control (empty vector-transfected) cells were seeded at a density of 7,000 cells/ml in 96-well plates. Cells were treated with IGF1 or dansylcadaverine or both, starting 24 hr after seeding for an additional 24 hr. Sample absorbance was measured with a spectrophotometer at a wavelength of 450–500 nanometers. Reference absorbance to measure non-specific readings was measured at a wavelength of 630–690 nanometers.

### Cell migration assays

Cell migration was monitored using scratch assays. MCF7, MCF10A, M12 and P69 cells were seeded aproxemtly 16 hr pre-scratch at a density of 20,000–30,000 cells/ml in a 96-well ImageLock microplate (Cat #4379, Essen BioScience, Ann Arbor, MI, USA). Scratch was done using WoundMaker^TM^ designed for this specific plate. Cells were treated with dansylcadaverine immediately after the scratch. The plates were placed into an IncuCyte ZOOM system for 48 hr, with repeat scanning every 2 hr. Analysis of the results was done using the IncuCyte ZOOM software.

### Statistical analysis

The statistical significance of differences was assessed by Student’s t-test (two samples, equal variance) or by ANOVA (post hoc Tukey HSD test) between multiple experimental groups. Results are presented as mean±SEM. A p-value of 0.05 was considered statistically significant.

## Results

### Nuclear translocation of IGF1R in non-malignant human cells

**Fig 1:**
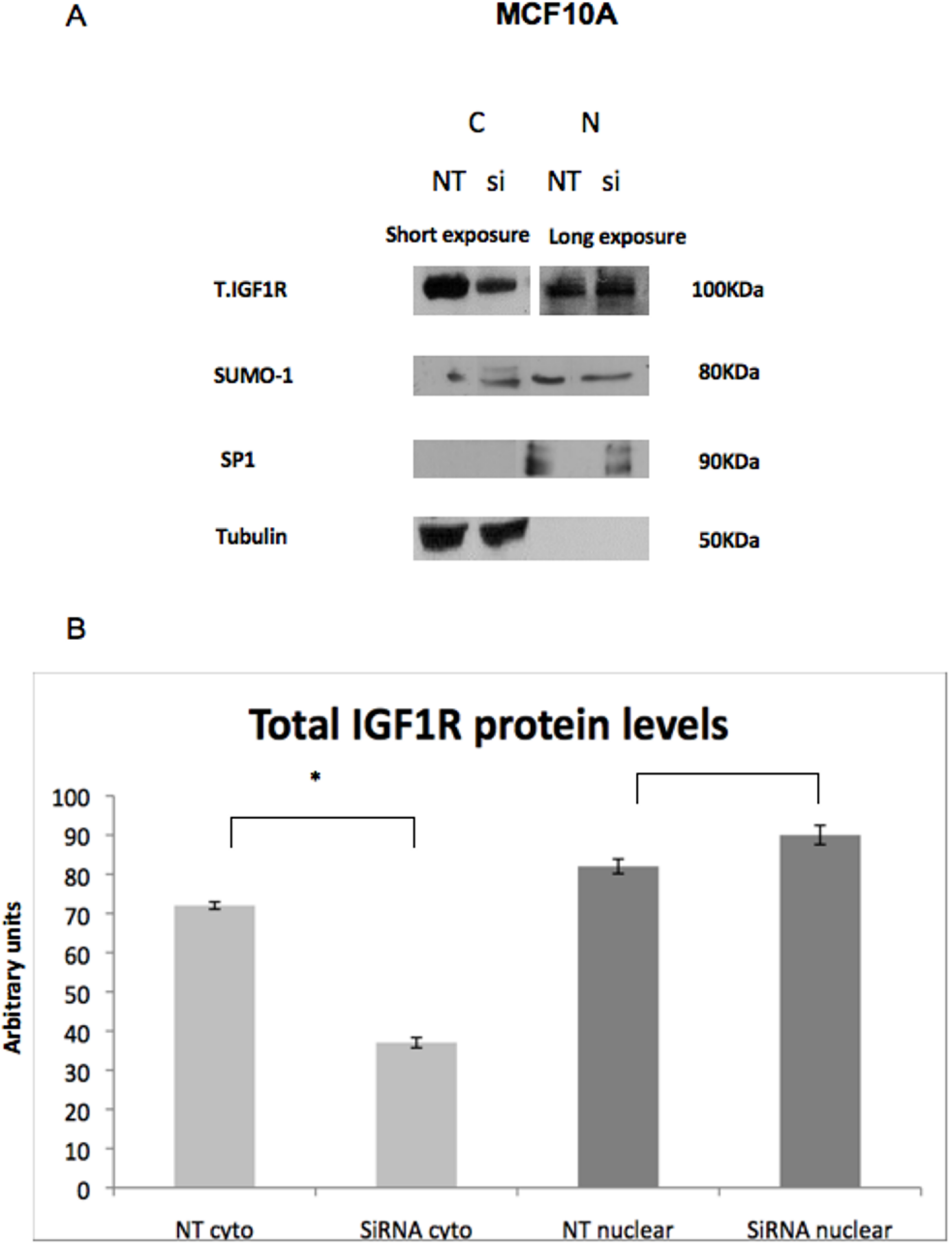
Western blot analysis of cytoplasmic and nuclear IGF1R in MCF10A cells. MCF10A cells were transfected with IGF1R siRNA (10 nM) or non-targeting (NT) siRNA and harvested 48 hr post transfection. (A) Western blot analysis of total IGF1R, Sumo-1, tubulin [a marker for the cytoplasmic **(C)** fraction] and SP1 [a marker for the nuclear **(N)** fraction]. (B) Densitometric analysis of Western blots. The results of a representative experiment repeated three times with similar results are shown. Analyses were done using the “ImageJ software”. Blots of the cytoplasmic fraction were exposed for short periods (2–5 min) whereas blots of the nuclear fraction were exposed for longer periods (10–15 min). Thus, it is not possible to compare between relative abundance of proteins between fractions.

The vast majority of studies conducted to date on nuclear IGF1R translocation were carried out in cancer-derived cells. Therefore, we set out to examine whether IGF1R nuclear transport occurs also in normal, non-transformed cells. For this purpose, fractionation of cytoplasmic and nuclear proteins was conducted in MCF10A cells, a non-malignant breast cell line, followed by Western blot analyses. IGF1R siRNA [or non-targeting (NT) siRNA] was used to evaluate the knockdown’s effect in the nucleus. Consistent with our hypothesis, IGF1R was present in the nuclear fraction of MCF10A cells (Fig 1). However, whereas IGF1R silencing was observed in the cytoplasmic fraction, no silencing was seen in the nuclear fraction. In addition, no differences in SUMO-1 levels were seen between the cytoplasmic and nuclear fractions, with or without IGF1R siRNA treatment.

### Confocal microscopy analysis of nuclear IGF1R

**Fig 2:**
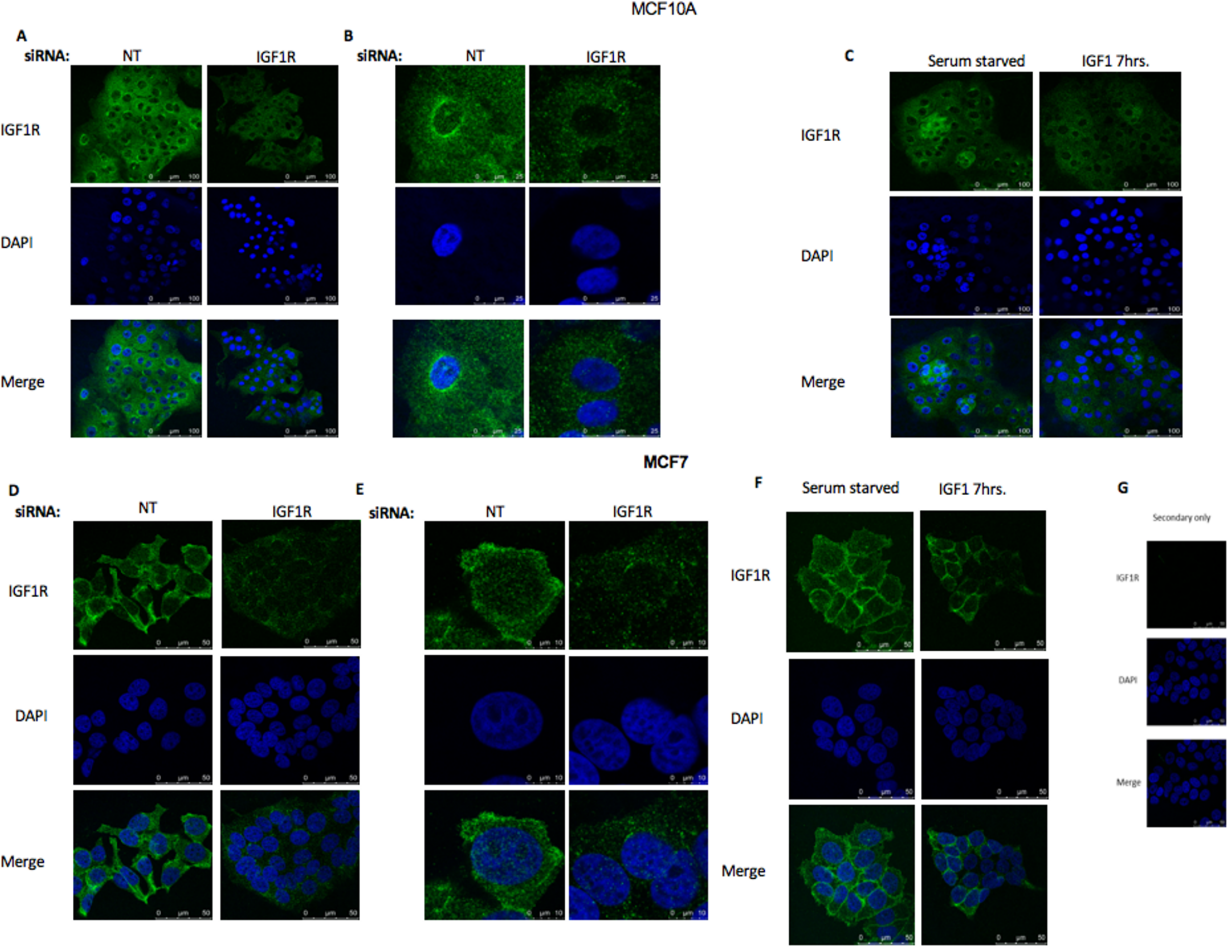
Confocal microscopy analysis of IGF1R nuclear localization. Confocal immunofluorescence microscopic imaging of IGF1R-expressing MCF10A **(A, B)** and MCF7 **(D, E)** cells. Cells were transfected with an IGF1R siRNA or NT siRNA for 48 h. **Effect of IGF1 treatment on IGF1R expression**. Serum-starved MCF10A **(C)** and MCF7 **(F)** cells were treated with IGF1 (50 ng/ml) for 7 hr, and IGF1R localization was evaluated by fluorescence imaging. Control experiment using only secondary antibody **(G).** Fixed cells were stained for IGF1R with a fluorescent donkey anti-rabbit antibody (green- 488) and DAPI (blue). Results of a representative experiment repeated three times with similar results are shown. Images **A-G** were photographed using x63NA1.4 amplification.

To corroborate the results of cell fractionation experiments showing a nuclear IGF1R localization in non-transformed cells, confocal microscopy analyses were performed in both benign MCF10A and malignant MCF7 breast cell lines. To this end, cells were transfected with IGF1R siRNA (or NT siRNA) for 48 hr. In parallel, cells were serum-starved for 24 hr, after which they were treated with IGF1 (50 ng/ml) for 7 hr. As shown in Fig 2A and 2D, IGF1R staining (green) was predominantly cytoplasmic but was also detectable in the nuclei of both cells. In control (NT) MCF10A cells, IGF1R was present also in perinuclear areas (Fig 2A and 2B). In MCF7 cells, however, we didn’t notice any IGF1R accumulation in perinuclear areas. Visual inspection of confocal images was confirmed by immuno-precipitation (IP) assays. In addition, a knockdown effect of the IGF1R siRNA compared to NT was noticed after 48 hr (Fig 2A and 2D). Treatment of both MCF10A and MCF7 cells with IGF1 for 7 hr led to a marked down-regulation of IGF1R, particularly at the perinuclear areas (Fig 2C). Therefore, data is consistent with the view that nuclear IGF1R translocation is a ligand-independent process (Fig 2C and 2F). No staining was detected in neither cell using only a secondary antibody (negative control- Fig 2G).

**Fig 3:**
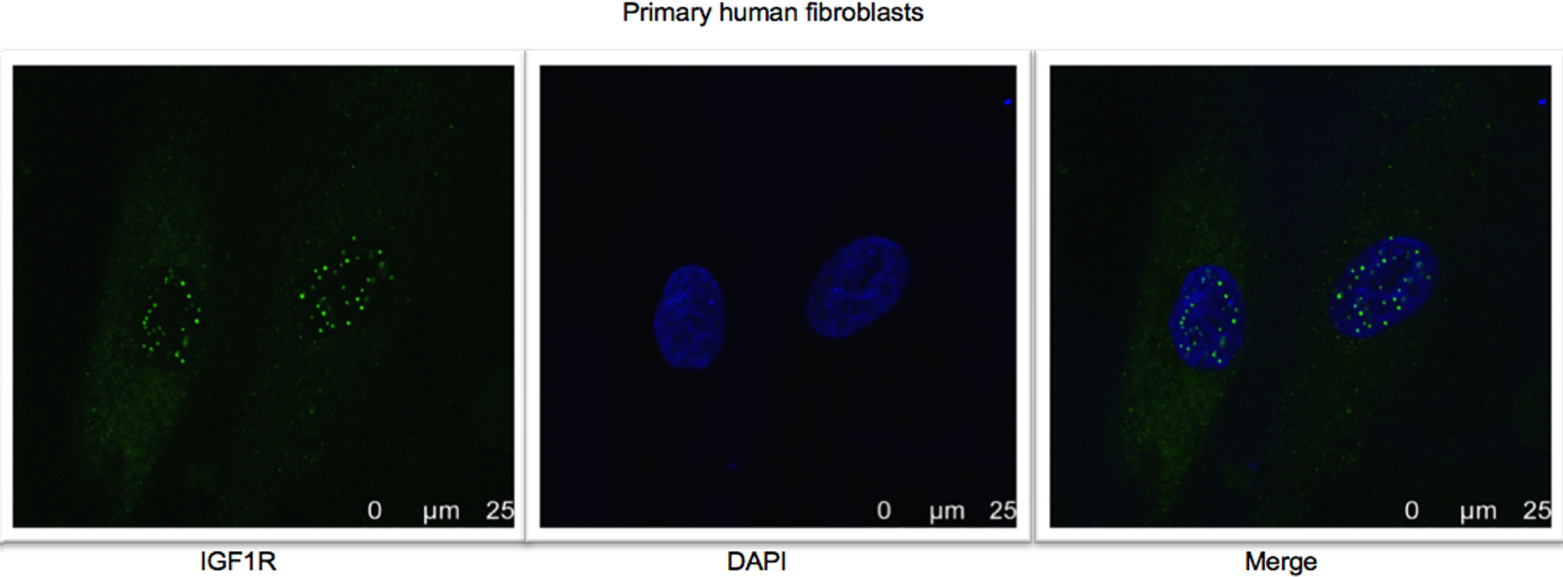
Confocal microscopy analysis of IGF1R nuclear localization in primary human fibroblasts. Fluorescence confocal microscope imaging of IGF1R in primary human fibroblasts. Cells were seeded in 24-well plates and after 24 hr were fixed and stained for IGF1R with a fluorescent donkey anti-rabbit antibody (green- 488) and DAPI (blue). Results of a representative experiment repeated two times with similar results are shown.

To further investigate the nuclear IGF1R translocation in an additional model of normal, non-transformed cells, microscope immunofluorescence assays were conducted on skin biopsies-derived primary human fibroblasts. The merged pictures show that IGF1R staining was predominantly cytoplasmic but was also detectable in the nuclear areas of fibroblasts (Fig 3).

### Effect of dansylcadaverine on nuclear IGF1R translocation

**Fig 4:**
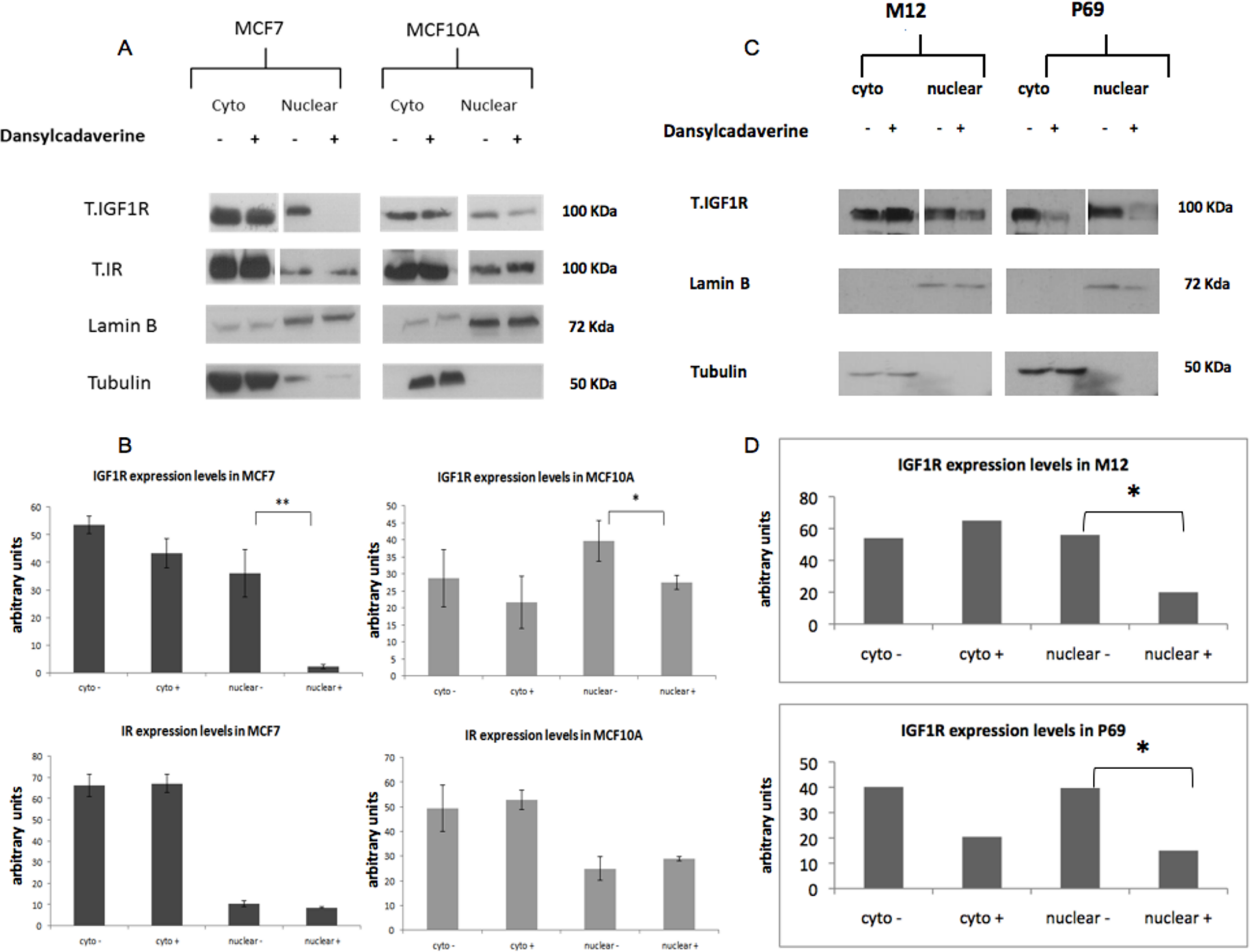
Effects of dansylcadaverine on IGF1R nuclear translocation in MCF7, MCF10A, M12 and P69 cells. Cells were treated with dansylcadaverine for 24 hr, after which cell fractionation was performed as described in Materials and Methods. **(A)** Western blot analysis of total IGF1R, total IR and tubulin (as a control for the cytoplasmic fraction) and lamin B1 (as a control for the nuclear fraction) in MCF7 and MCF10A cells. Given that different exposure times were carried out for the cytoplasmic and nuclear fractions, it is not possible to compare expression levels between both fractions. **(B)** Quantitative analysis of the results. Analyses were done using “ImageJ” software. **(C)** Western blot analysis of total IGF1R, tubulin and lamin B1 in M12 and P69 cells. **(D)** Quantitative analysis of the results. Results of a representative experiment repeated three times with similar results are shown. The “-“and “+” symbols represent control and dansylcadaverine-treated cells, respectively.

To assess the impact of nuclear IGF1R on biological processes and their potential relevance to tumorigenesis, we inhibited IGF1R translocation into the nucleus by treating MCF7 and MCF10A cells with dansylcadaverine for 24 hr. Dansylcadaverine was proven to inhibit the translocation of several proteins, including IGF1R, into the nucleus [14]. The mechanism of action of dansylcadaverine involves inhibition of clathrin, which has a key role in nuclear endocytosis. After treatment with dansylcadaverine there was a major reduction in total IGF1R levels in the nuclear fractions of both MCF7 and MCF10A cells, compared to untreated cells (Fig 4A). Of notice, the effect of the inhibitor was significantly stronger in MCF7 cells (Fig 4B). Despite the fact that dansylcadaverine is generally regarded as a non-specific endocytosis inhibitor, we examined its effect also on insulin receptor (IR) translocation. The rationale for this experiment was the very high structural homology between IR and IGF1R (~80%) (20). Of interest, no marked decrease in IR levels upon dansylcadaverine treatment was seen in neither cell line. These results may suggest that IGF1R and IR employ different internalization pathways.

In addition to breast cancer cells, the impact of nuclear IGF1R was also assessed in two prostate cell lines, P69 and M12. P69 is a non-tumorigenic prostate epithelial-derived cell line whereas the M12 line is a metastatic derivative of P69. Nuclear translocation was inhibited by treating the cells with dansylcadaverine for 24 hr. Western blots showed a major reduction in total IGF1R levels in the nuclear fractions of both cells compared to untreated control cells (Fig 4C).

### Effects of nuclear IGF1R transport inhibition on cell proliferation

**Fig 5:**
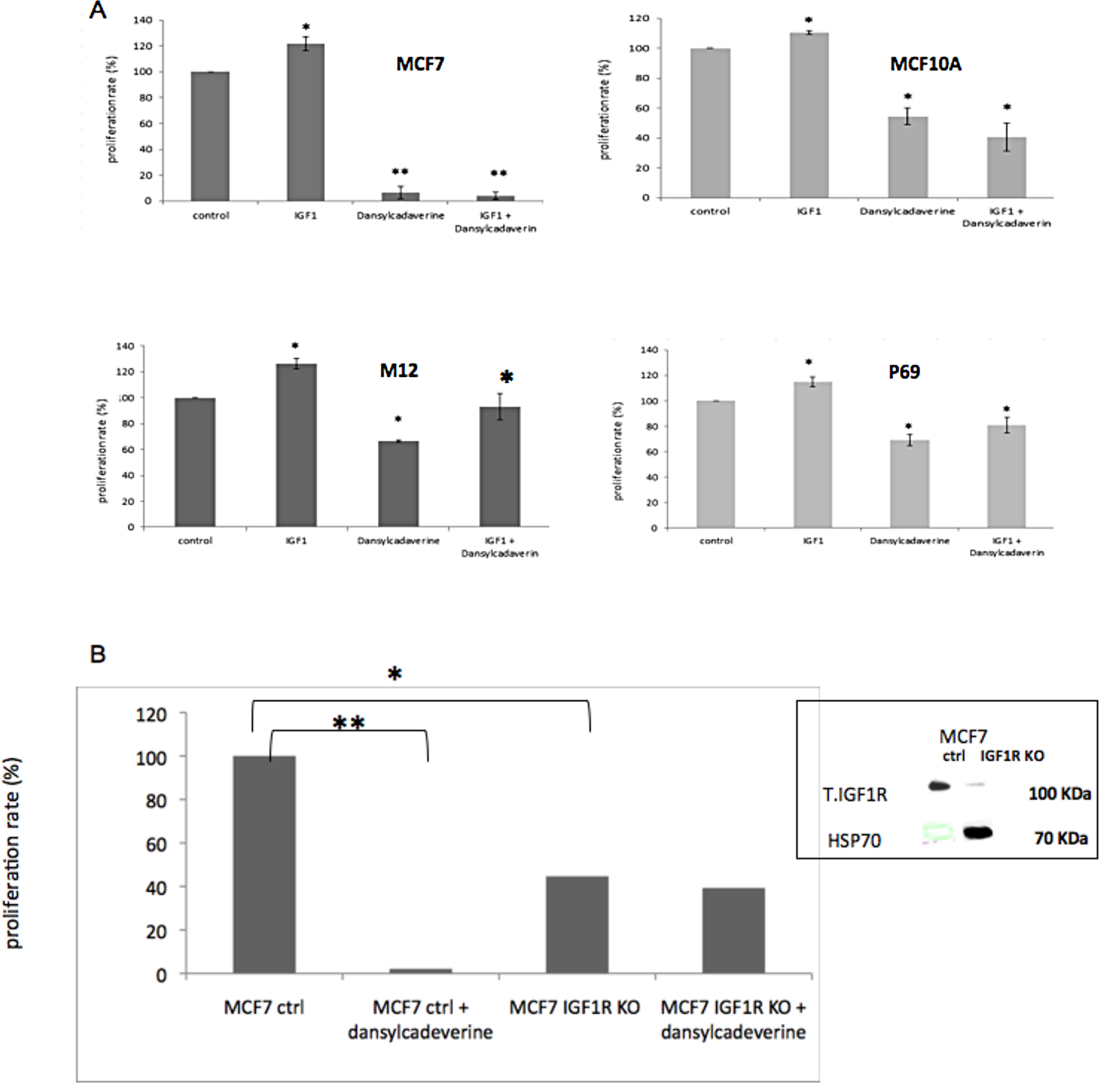
Effects of dansylcadaverine on cell proliferation. (A) MCF7, MCF10A, M12 and P69 cells were treated with dansylcadaverine, with or without IGF1, for 24 hr, after which absorbance was measured. (B) Stable MCF7/IGF1R KO and empty vector-transfected (control) cells were treated with dansylcadaverine for 24 hr, after which absorbance was measured. (*) significantly different versus control. Results of a representative experiment repeated three times with similar results are shown. The inset depicts the basal IGF1R levels in IGF1R-silenced and control (empty vector-transfected) cells. Heat shock protein-70 (hsp70) was used as a loading control).

To assess the potential impact of IGF1R nuclear translocation inhibition on cell growth, proliferation assays were conducted in dansylcadaverine-treated cells using an XTT cell proliferation kit. MCF7 and MCF10A cells were treated with the inhibitor, with or without IGF1 treatment, for 24 hr, after which absorbance was measured. As expected, IGF1 had a small, but significant, proliferative effect in both cell lines (Fig 5A). In addition, dansylcadaverine-treated cells display a lower proliferation rate in comparison to untreated cells. However, the effect of dansylcadaverine in malignant MCF7 cells was significantly more potent than in benign MCF10A cells (~90% decrease compared to 40% decrease). This could be explained by the stronger dansylcadaverine effect on IGF1R nuclear translocation noticed in MCF7 cells (Fig 4). However, treatment of both MCF7 and MCF10A cells with both IGF1 and dansylcadaverine had no compensatory effect on proliferation rate.

To evaluate the general nature of IGF1R nuclear transport, the impact of dansylcadaverine was also investigated in the P69 and M12 prostate cancer cell lines. Dansylcadaverine led to a reduction in proliferation in both cell lines. Of interest, unlike MCF7 and MCF10A cells, treatment with both IGF1 and dansylcadaverine had a compensatory effect compared to dansylcadaverine alone (Fig 5A). We speculate that this compensatory effect in P69 and M12 cells is due to the lower IGF1R levels in these cells.

To evaluate the specificity of dansylcadaverine as an IGF1R nuclear translocation inhibitor, proliferation assays were conducted using stable MCF7 cells with a silenced IGF1R (MCF7/IGF1R KO) and empty vector-transfected (control) cells (Fig 5B, inset). Cells were treated with dansylcadaverine after which XTT proliferation assays were performed. As shown in Fig 5B, basal proliferation was reduced in MCF7/IGF1R KO cells (compare bars 1 to 3). As expected, dansylcadaverine markedly reduced proliferation rate in empty vector-transfected MCF7 cells (compare bars 1 to 2). However, the inhibitor had no effect in stable MCF7/IGF1R KO cells (compare bars 3 to 4).

### Effects of nuclear IGF1R transport inhibition on cell migration

**Fig 6:**
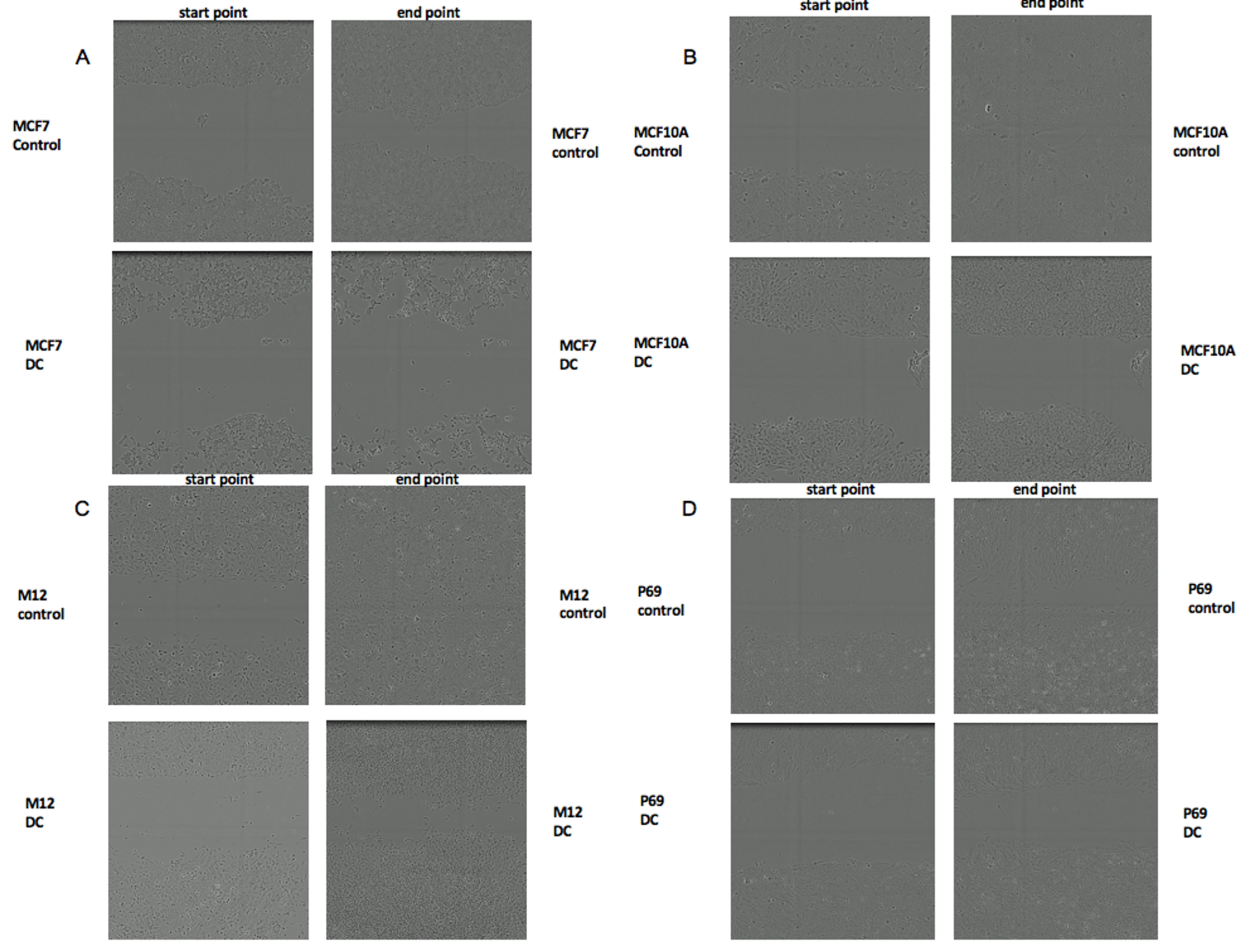
Effects of dansylcadaverine on cell migration of MCF7, MCF10A, M12 and P69 cells. A scratch was done using WoundMaker^TM^ in MCF7 (A), MCF10A (B), M12 (C) and P69 (D) cells. Then, cells were treated with dansylcadaverine for 48 hr, and scanned and photographed every two hours. Representative micrographs taken at start and end points (control vs. dansylcadaverine treatment), are shown. Results were in triplicates and every wound was photographed in two different areas with similar results are shown.

To evaluate the impact of dansylcadaverine on cell migration, scratch assays were conducted. A scratch was done using WoundMaker^TM^ in MCF7, MCF10A, M12 and P69 cells. Then, cells were treated with dansylcadaverine for 48 hr and cells were scanned and photographed at the scratch (wound) area every two hours. As seen in Fig 6A–6D, the scratch area after treatment with dansylcadaverine was wider at the end point in all cell types, compared to controls. These results indicate that blocking IGF1R nuclear migration by dansylcadaverine led to lower migration rates (Fig 6). As in proliferation assays, the most potent effect of dansylcadaverine was seen in MCF7 cells (Fig 6A).

### Combined effect of nuclear IGF1R transport and IGF1R inhibitors

**Fig 7:**
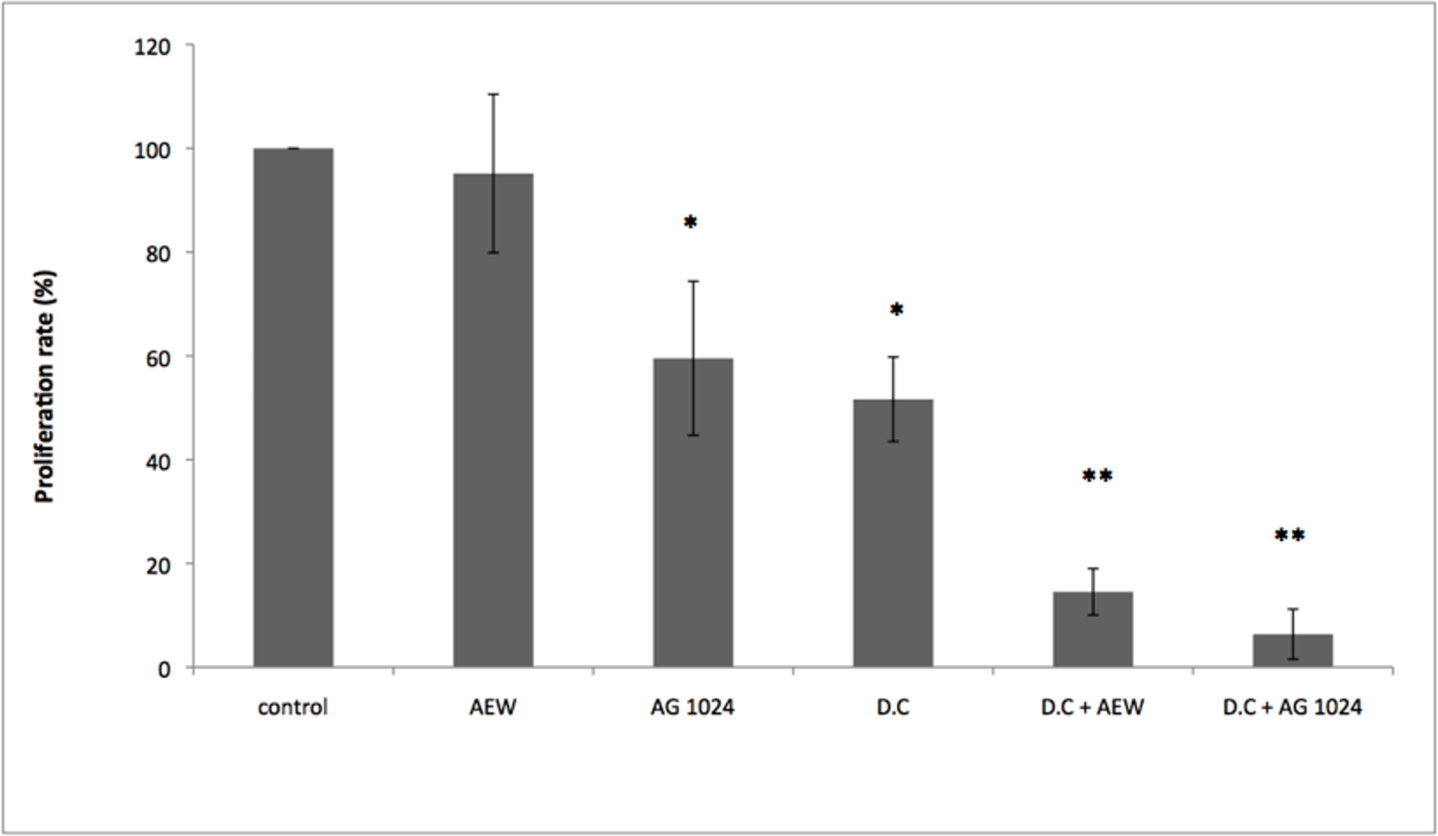
Effects of dansylcadaverine and IGF1R inhibitors on cell proliferation of M12 cells. M12 cells were treated with dansylcadaverine, or with the selective IGF1R inhibitors AEW541 or AG1024, or both dansylcadaverine and IGF1R inhibitors for 24 hr, after which absorbance was measured. (*) significantly different versus control. Results of a representative experiment repeated three times with similar results are shown.

The IGF1R emerged in recent years as a potential therapeutic target in oncology [16–18]. However, most clinical trials led to mixed results. There is, therefore, a critical need to define predictive biomarkers in order to identify patients who may benefit from IGF1R-directed therapies [19]. The impact of nuclear IGF1R presence on the outcome of IGF1R targeted therapies has not yet been systematically explored [11]. Therefore, we evaluated the effect of dansylcadaverine along with the selective IGF1R tyrosine kinase inhibitors AEW541 or tyrphostin AG1024 on cell proliferation in the M12 prostate cancer cell line. As shown in Fig 7, treatment with AEW541 did not have a marked inhibitory effect on cell proliferation whereas treatment with AG1024 led to a significant decrease in proliferation. As described above, treatment with dansylcadaverine had a significant effect on proliferation. Finally, treatment with AEW541 or AG1024 along with dansylcadaverine had marked synergistic effects in proliferation rates (AEW541 + dansylcadaverine: ~85% inhibition versus ~5% by AEW541 alone; AG1024 + dansylcadaverine: ~95% inhibition versus ~40% by AG1024 alone) (p-value of 0.01).

## Discussion

The role of the IGF axis in normal growth and differentiation as well as in pathological conditions has been well established. The classical model for insulin-like hormones action involves the ligand-induced phosphorylation of a heterotetrameric cell-surface tyrosine kinase receptor, with ensuing activation of cytoplasmic signaling cascades. The recent identification of nuclear IGF1R translocation provides an additional level of biological regulation by allowing a typical transmembrane receptor to function in a discrete, membrane-bound cellular environment.

Using cell fractionation, confocal microscopy and additional cellular and biochemical analyses, we and others have previously shown that IGF1R is present in the nucleus of breast and other cancer cell lines and tissues. Specifically, cell-surface IGF1R undergoes modification by SUMO-1, with ensuing translocation to the nucleus. Evidence was further provided showing that nuclear IGF1R functions as a transcriptional activator. In this context, the nuclear receptor interacts with a number of co-activators *via* protein-protein interactions. Furthermore, nuclear IGF1R was also shown to bind genomic DNA in, apparently, a sequence-specific manner. Among other target genes, IGF1R binds and autoregulates its cognate promoter at the transcriptional level [12].

While most studies conducted to date were performed in cancer-derived cell lines, data presented here indicate that nuclear IGF1R translocation may also occur in normal, non-transformed cells, including primary diploid fibroblasts and immortalized non-malignant mammary gland-derived cells (e.g., MCF10A). Hence, nuclear IGF1R appears to fulfill a physiological, not only pathological, role in the context of IGF1 signaling. Using both immunofluorescence and cell fractionation techniques we identified the IGF1R in the nucleus of both non-carcinogenic MCF10A and carcinogenic MCF7 human breast cancer cell lines. In addition, we noticed the accumulations of IGF1R in perinuclear areas of MCF10A cells in addition to the cytoplasm and nucleus. Furthermore, we demonstrated in cell fractionation assays that siRNA against IGF1R led to a decrease in IGF1R levels in the cytoplasmic, but not nuclear, fraction. A potential explanation for this differential effect relates to the mechanism of action of siRNA, which involves mRNA degradation in the cytoplasm, but not in the nucleus. Therefore, siRNA is expected to be less effective in nucleus. Alternatively, we cannot discard the possibility that Western blots have a reduced sensitivity that doesn’t allow them to detect the siRNA effect. We also evaluated SUMO-1 levels both in the cytoplasmic and nuclear fractions (Fig 1). However, we were unable to detect any major difference between the fractions with or without siRNA transfection.

The question whether SUMOylation is an obligatory prerequisite for nuclear translocation remains a controversial issue. Thus, whereas Sehat et al reported that SUMOylation is a prerequisite for the nuclear translocation of IGF1R [13], other investigators have provided data showing that SUMOylation is not a critical requisite [14]. Thus, Lin et al showed that SUMOylation is important for IGF1R-induced cell proliferation but is not an absolute requirement for nuclear translocation [20]. In addition, Deng et al has established a cell model that prevents IGF1R from being SUMOylated and demonstrated that over-accumulated nuclear IGF1R leads to high expression of SUMO-conjugating enzyme Ubc9 [21]. Finally, the issue whether receptor internalization is a ligand-dependent process has not yet been solved. While Aleksic et al [14] showed that IGF1-stimulated IGF1R activation is indeed required for nuclear translocation, our present data indicates that nuclear IGF1R transport may occur in a ligand-independent manner.

We showed that treatment with dansylcadaverine inhibited IGF1R nuclear translocation in breast-derived -MCF7 and MCF10A- and prostate-derived–M12 and P69-cells lines. In addition, inhibition of nuclear IGF1R translocation with dansylcadaverine had a significant effect on proliferation and migration. We assume that nuclear IGF1R interacts with other proteins linked to cell proliferation or/and migration. The molecular basis of these protein-protein interactions is the focus of current investigation. Consistent with subcellular localization data, the biological effect of the inhibitor was particularly strong in malignant MCF7 cells. Furthermore, the effect of dansylcadaverine on proliferation was stronger in IGF1R-expressing cells than in cells with a silenced IGF1R (Fig 5B). Of notice, the fact that dansylcadaverine had no effect on nuclear IR levels might be consistent with the notion that IGF1R and IR employ different pathways for nuclear translocation.

As mentioned above, the IGF1R emerged in recent years as a potential therapeutic target in oncology. Our results showing a synergistic effect of a nuclear translocation blocker along with selective IGF1R inhibitors may be of translational relevance (Fig 7). Of importance, the possibility of additional, non-IGF1R mediated effects of dansylcadaverine cannot be entirely excluded. Consistent with our data, Asmane et al showed that cellular IGF1R localization might have an impact on the efficacy of IGF1R inhibitors [22]. Specifically, patients with advanced sarcoma achieved prolonged free survival upon IGF1R monoclonal antibody therapy when IGF1R was present only in the nucleus compared to patients with mixed IGF1R localization. Taken together, nuclear IGF1R may serve as: (1) predictive biomarker; and (2) therapeutic target for cancer.

In summary, nuclear transport of cell-surface receptors constitutes a novel regulatory mechanism that may provide an additional layer of biological regulation. Our data is consistent with the notion that nuclear IGF1R fulfills important physiological as well as pathological roles. Future studies will be aimed at identifying pathways and proteins that interact with nuclear IGF1R.
